# The value of indocyanine green-FLOW800 in microvasculature for predicting cerebral hyperperfusion syndrome in moyamoya disease patients

**DOI:** 10.1038/s41598-023-45676-1

**Published:** 2023-10-26

**Authors:** Zhongxiang Guo, Zhaohui Yan, Fan Qu, Dekui Cheng, Chao Wang, Yugong Feng

**Affiliations:** 1 Department of Neurosurgery, The Affiliated Hospital of Qingdao University, Jiangsu Road No. 16, Qingdao, 266003 Shandong Province China; 2 Department of Neurosurgery, Haiyang People’s Hospital, Haiyang Road No. 37, Haiyang, 265199 Shandong Province China; 3 Department of Neurosurgery, Liaocheng People’s Hospital, Dongchang West Road No. 67, Liaocheng, 25200 Shandong Province China

**Keywords:** Medical research, Neurology

## Abstract

Among the notable complications of direct hemodynamic reconstruction for moyamoya disease (MMD) is cerebral hyperperfusion syndrome (CHS). In this study, we evaluated hemodynamic changes in small regional microvasculature (SRMV) around the anastomosis site by using indocyanine green (ICG)-FLOW800 video angiography and verified that it better predicted the onset of CHS. Intraoperative ICG-FLOW800 analysis was performed on 31 patients (36 cerebral hemispheres) with MMD who underwent superficial temporal artery-middle cerebral artery (MCA) bypass grafting at our institution. The regions of interest were established in the SRMV and thicker MCA around the anastomosis. Calculations were made for half-peak to time (TTP_1/2_), cerebral blood volume (CBV), and cerebral blood flow (CBF). According to the presence or absence of CHS after surgery, CHS and non-CHS groups of patients were separated. The results showed that ΔCBV and ΔCBF were substantially greater in SRMV than in MCA (*p* < 0.001). Compared with the non-CHS group, ΔCBF and ΔCBV of SRMV and MCA were considerably greater in the CHS group (*p* < 0.001). ΔCBF and ΔCBV on the ROC curve for both SRMV and MCA had high sensitivity and specificity (SRMV: ΔCBF, AUC = 0.8586; ΔCBV, AUC = 0.8158. MCA: ΔCBF, AUC = 0.7993; ΔCBV, AUC = 0.8684). ICG-FLOW800 video angiography verified the differential hemodynamic changes in the peri-anastomotic MCA and SRMV before and after bypass surgery in patients with MMD.

## Introduction

Moyamoya disease (MMD) is a rare chronic ischemic cerebrovascular disease of unknown etiology, characterized by progressive narrowing or occlusion of the terminal internal carotid arteries and the development of an abnormal vascular network around it. On imaging, this compensatory creation of an aberrant blood vessel network resembles a puff of smoke, hence the name “smoke vessels”^1–3^. These compensatory “smoke vessels” are extremely fragile, with the potential to rupture, bleed, or cause transient cerebral ischemia. Direct revascularization strategies, like the anastomosis between the superficial temporal artery and middle cerebral artery (STA-MCA), can decrease the risk of cerebral hemorrhage and ischemia significantly^4–9^. A risky complication of MMD bypass surgery, cerebral hyperperfusion syndrome (CHS), can lead to severe headaches, aphasia, seizures, focal neurological deficits, and even intracranial hemorrhage. Although the final outcome of these patients was mostly good, the early prediction of post-operative CHS is of great interest because the management of hyperperfusion is contradictory to the management of ischemia^10–16^. Many studies have confirmed the hemodynamic changes following bypass surgery, and this hemodynamic dysfunction appears to be a predictor of CHS^17–20^.

Indocyanine green (ICG) is a fluorescent imaging agent that can be safely used in clinical trials, and surgical microscopes equipped with ICG fluorescence angiography can capture images of the developing cerebral vessels^21,22^. The FLOW800 (Zeiss Meditec, Oberkochen, Germany) color fluorescence angiography is a technical upgrade on the ICG fluorescence angiography. By capturing information on the parameters of the ICG fluorescence angiography and analyzing it post-processed by the FLOW800 software integrated in the operating microscope, it provides a visually delayed color map of the hemodynamic and perfusion changes, which reflects the intraoperative cerebral perfusion by semi-quantitative assessment of the hemodynamic changes in the region of interest (ROI)^23–25^.

**Figure 1:**
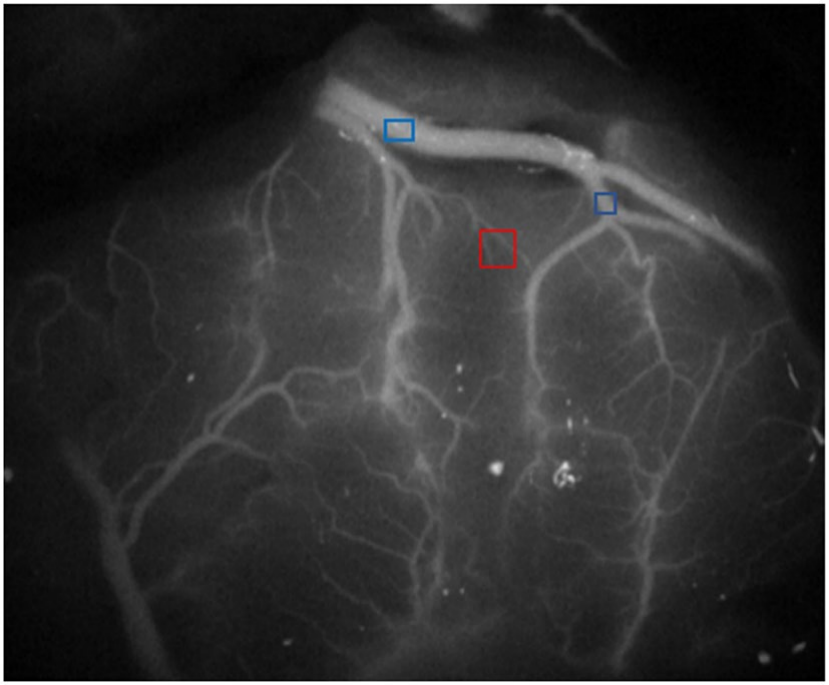
Distinction between the two types of ROI. The ROI represented by the red box is SRMV, and the ROIs represented by the blue boxes are MCA.

The vast majority of international reports on predicting CHS after MMD by ICG-FLOW800 video angiography currently place the selection of the ROI on the surgically anastomosed MCA and other surrounding thicker vessels^20,26^. In this study, we shifted the ROI selection target to the microvasculature. We refer to the terminal branches of the vessels surrounding the receptor arteries in a small area as “small regional microvasculature” (SRMV) (Fig. 1). Our objectives were to assess the hemodynamic changes in the peri-anastomotic MCA and SRMV before and after bypass surgery using ICG-FLOW800 video angiography, to verify whether the hemodynamic changes in SRMV could be used as a predictor of CHS after direct hemodynamic reconstruction for MMD, and to calculate and compare the diagnostic accuracy of the MCA and SRMV for predicting CHS separately.

## Results

### Demographics

One of the 36 cerebral hemispheres had a new cerebral infarction after surgery. This 1 hemisphere (1 patient) was excluded from the study. With a mean age of 51.97 years and a range of 15–72 years, this study included 35 cerebral hemispheres (12 hemispheres in 10 patients who were male and 23 hemispheres in 21 patients who were female) (Table 1). For the CHS group, 16 of the 35 hemispheres (45.7%) showed symptoms of hemodynamic disturbances, such as aphasia, seizures and limb numbness, after revascularization. The non-CHS group included the remaining 19 cases (54.3%), which presented with asymptomatic or mild headaches. Between the two groups, there were no statistically differences in terms of gender, age, or surgical side (*p* > 0.05).

**Table 1:** Comparison of clinical parameters between groups with and without CHS.

Parameter	CHS	Non-CHS	*p* value
Hemisphere	16	19	
Mean age (years)	52.50 ± 10.09	51.53 ± 16.56	0.832
Sex			
Male (%)	4 (25.0)	8 (42.1)	0.297
Female (%)	12 (75.0)	11 (57.9)	
Operated side			
Right (%)	8 (50.0)	13 (68.4)	0.379
Left (%)	8 (50.0)	6 (31.6)	

Values are numbers of patients or hemispheres unless otherwise indicated. Mean values are given with SDs.

### Hemodynamic data discrepancies between groups

**Figure 2:**
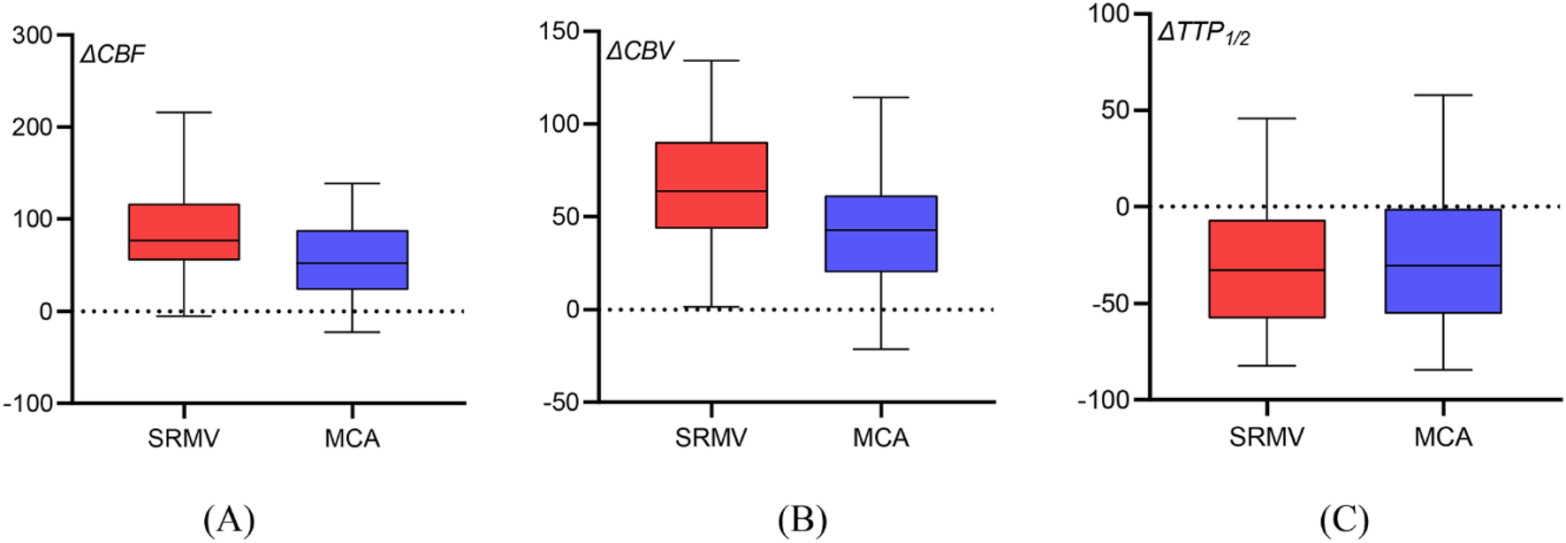
Analysis of hemodynamic parameters in the SRMV group and MCA group. Compared to the MCA group, both ΔCBF (86.99% vs. 54.61%, *p* < 0.001) and ΔCBV (67.82% vs. 42.69%, *p* < 0.001) in the SRMV group were substantially greater, and ΔTTP_1/2_ was slightly lower (− 28.07% vs. − 25.58%, *p* = 0.061).

**Figure 3:**
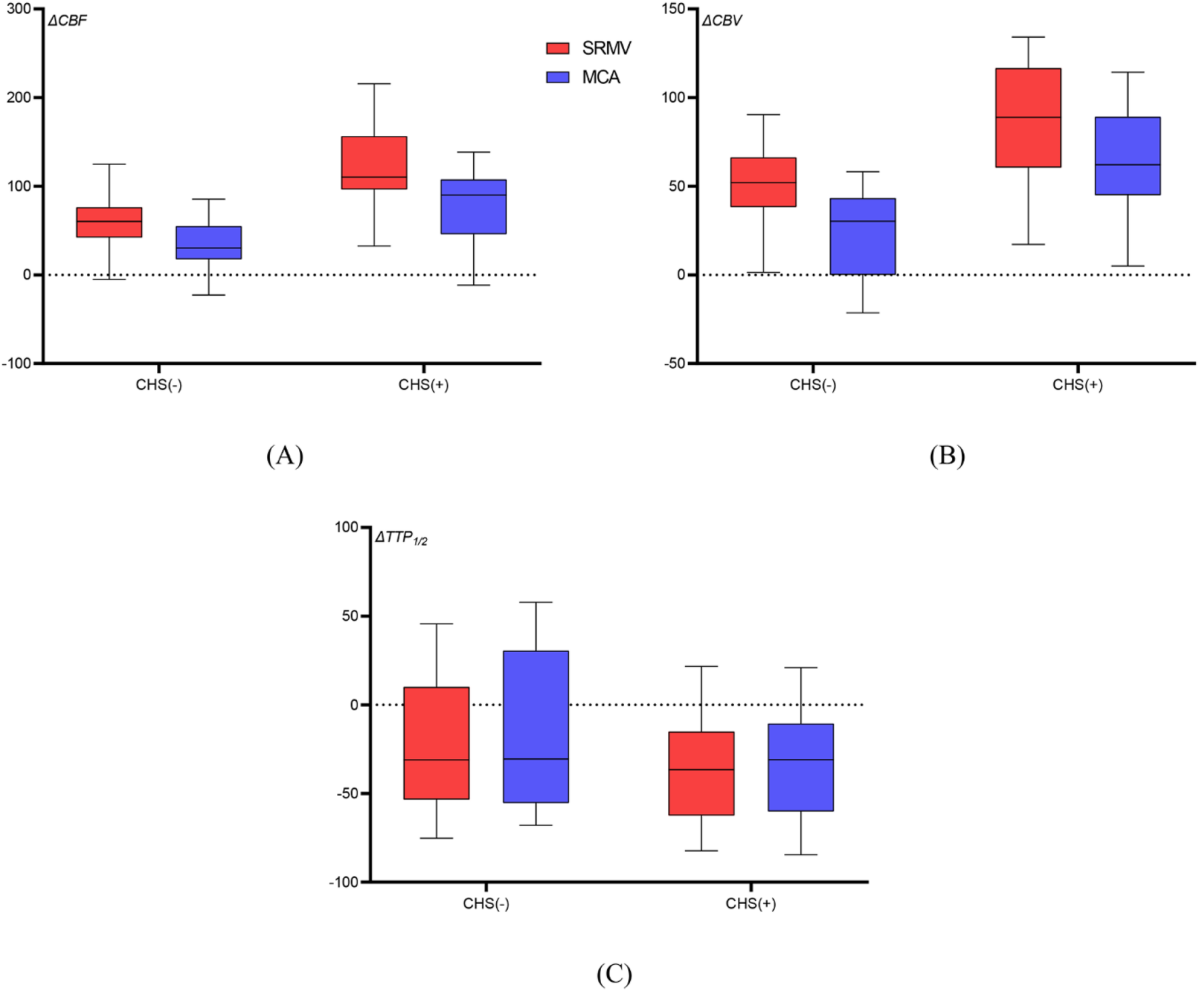
Subgroup analyses of hemodynamic parameters were performed in the CHS and non-CHS groups. Compared to the non-CHS group, ΔCBF (SRMV, 119.51% vs. 59.62%, *p* < 0.01; MCA, 77.69% vs. 35.17%, *p* < 0.001) and ΔCBV (SRMV, 87.89% vs. 50.91%, *p* < 0.01; MCA, 65.59% vs. 23.41%, *p* < 0.001) were substantially greater in both the SRMV and MCA groups in the CHS group and, conversely, ΔTTP_1/2_ (SRMV, − 36.46% vs. − 21.01%, *p* = 0.199; MCA, − 33.56% vs. − 18.85%, *p* = 0.251) was slightly lower.

Comparisons were made between the hemodynamic data from the SRMV and MCA groups, as well as the CHS and non-CHS groups (Figs. 2 and 3). In the SRMV group, the values of the hemodynamic parameters were as follows: ΔCBF, 86.99% ± 50.03%; ΔTTP_1/2_, − 28.07% ± 35.16%; and ΔCBV, 67.82% ± 34.14%. In the MCA group, the values of the hemodynamic parameters were as follows: ΔCBF, 54.61% ± 42.73%; ΔTTP_1/2_, − 25.58% ± 37.33%; and ΔCBV, 42.69% ± 34.21%. Compared to the MCA group, both ΔCBF and ΔCBV were significantly higher in the SRMV group (*p* < 0.001) and ΔTTP_1/2_ was slightly lower (*p* = 0.061). The hemodynamic parameter values in the SRMV for the CHS group were as follows: ΔCBF, 119.51% ± 45.66%; ΔTTP_1/2_, − 36.46% ± 28.98%; and ΔCBV, 87.89% ± 33.62%. Similarly, the values of the hemodynamic parameters in the MCA were as follows: ΔCBF, 77.69% ± 45.09%; ΔTTP_1/2_, − 33.56% ± 30.72%; and ΔCBV, 65.59% ± 29.48%. The hemodynamic parameter values in the SRMV for the non-CHS group were as follows: ΔCBF, 59.62% ± 35.48%; ΔTTP_1/2_, − 21.01% ± 38.98%; and ΔCBV, 50.91% ± 24.48%. Similarly, the values of the hemodynamic parameters in the MCA were as follows: ΔCBF, 35.17% ± 29.69%; ΔTTP_1/2_, − 18.85% ± 41.73%; and ΔCBV, 23.41% ± 25.04%. The ΔCBF and ΔCBV were significantly higher in the SRMV and MCA of the CHS group compared to the non-CHS group (*p* < 0.001), while TTP_1/2_ was slightly lower (*p* = 0.199, *p* = 0.251).

### ROC analysis of hemodynamic parameters

**Figure 4:**
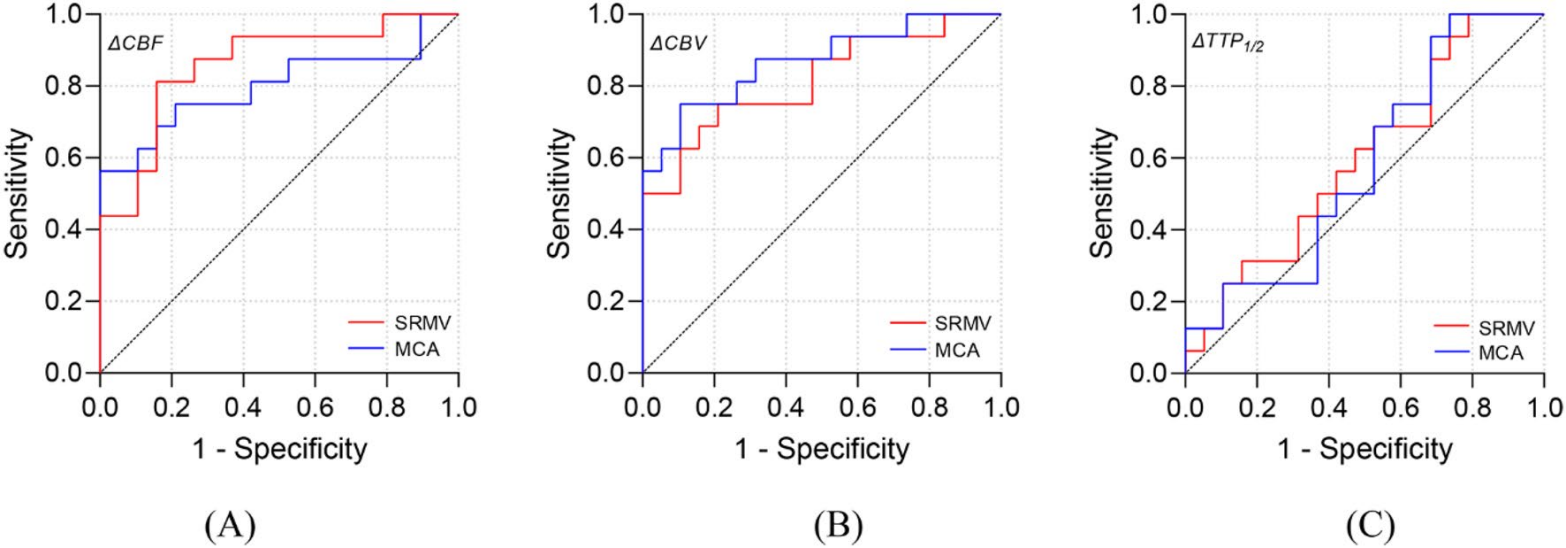
ROC curve analysis for hemodynamic parameters in the SRMV group and MCA group. The results showed that ΔCBF in the SRMV group and ΔCBV in the MCA group had relatively high diagnostic accuracy (SRMV group: ΔCBF, AUC = 0.8586, 95% CI 0.7311–0.9860, *p* = 0.0003; ΔCBV, AUC = 0.8158, 95% CI 0.6700–0.9616, *p* = 0.0015. MCA group: ΔCBF, AUC = 0.7993, 95% CI 0.6378–0.9608, *p* = 0.0026; ΔCBV, AUC = 0.8684, 95% CI 0.7462–0.9907, *p* = 0.0002). Conversely, the diagnostic accuracy of ΔTTP_1/2_ in both groups was low (AUC = 0.5987, AUC = 0.5822).

The ROC curves were used to analyze each parameter associated with postoperative CHS in the SRMV and MCA groups, respectively (Fig. 4). ΔCBF in the SRMV group had an ideal cutoff of 95.04% (sensitivity: 81.25%, specificity: 84.21%, Youden’s index: 0.6546) with an AUC of 0.8586 (95% CI 0.7311–0.9860, *p* = 0.0003) for identifying patients with CHS. With an ideal threshold of 66.99% (sensitivity: 75.00%, specificity: 78.95%, Youden’s index: 0.5395), the AUC was 0.8158 (95% CI 0.6700–0.9616, *p* = 0.0015) for the ΔCBV. In contrast, the AUC was 0.5987 (95% CI 0.4095–0.7879, *p* = 0.3205) for the ΔTTP_1/2_, which was not sufficiently accurate for diagnosis. ΔCBF in the MCA group had an ideal cutoff of 86.77% (sensitivity: 56.25%, specificity: 100%, Youden’s index: 0.5625) with an AUC of 0.7993 (95% CI 0.6378–0.9608, *p* = 0.0026) for identifying patients with CHS. With an ideal threshold of 49.24% (sensitivity: 75.00%, specificity: 89.47%, Youden’s index: 0.6447), the AUC was 0.8684 (95% CI 0.7462–0.9907, *p* = 0.0002) for the ΔCBV. In contrast, the AUC was 0.5822 (95% CI 0.3901–0.7744, *p* = 0.4078) for the ΔTTP_1/2_, which was not sufficiently accurate for diagnosis.

### Typical examples

**Figure 5:**
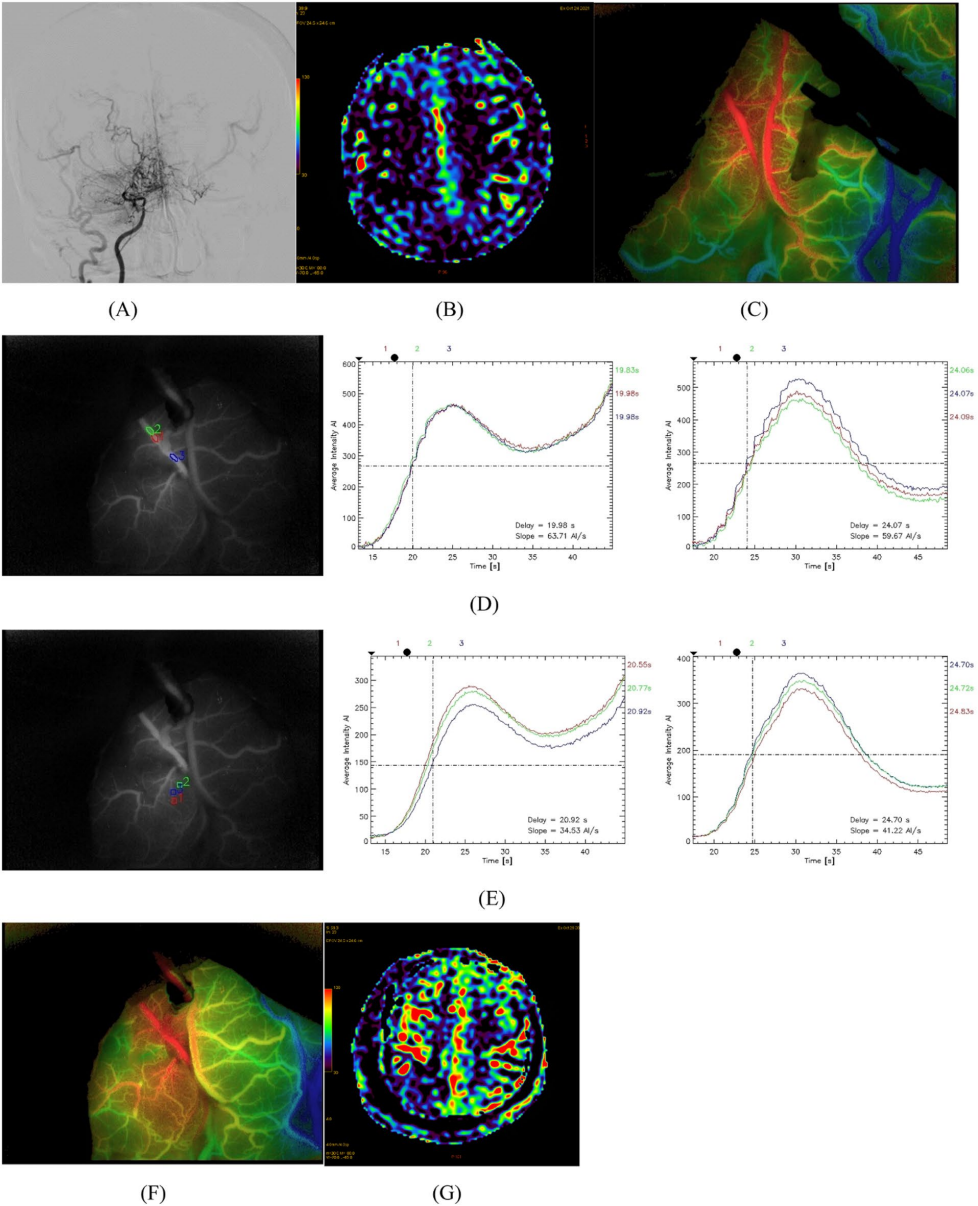
DSA showed occlusion of the right ACA and MCA with moyamoya vessel formation (**A**). ASL showed hypoperfusion in the right hemisphere (**B**). Suitable receptor vessels were selected by ICG-FLOW800 (**C**). ROIs were set up, and hemodynamic analyses were performed in the SRMV and the thick MCA around the anastomosis, respectively (**D**,**E**). ICG-FLOW800 showed good hematologic reconstruction (**F**). On the second postoperative day, the patient developed aphasia, and the magnetic resonance perfusion imaging showed increased in perfusion compared to the preoperative period (**G**).

Our hospital received a lady of 51 years who had been experiencing headaches and limb weakness for a year. A preoperative magnetic resonance perfusion imaging examination showed hypoperfusion in the right hemisphere. DSA shows occlusion of the right anterior cerebral arteries (ACA) and MCA with peripheral moyamoya vessel formation. After ruling out any surgical contraindications, a STA-MCA bypass was carried out on the right side. Intraoperatively, ROIs were set at SRMV and MCA by ICG-FLOW800 video angiography, respectively. Analysis of the hemodynamic parameters of the two different ROIs showed that ΔCBF and ΔCBV increased more significantly in SRMV than in MCA. On the second postoperative day, the patient had aphasia. The postoperative magnetic resonance perfusion imaging revealed that right cerebral perfusion had significantly increased since the preoperative period. (Fig. 5).

## Discussion

Our analysis using ICG-FLOW800 showed significant differences in ΔCBF and ΔCBV between the SRMV and MCA groups. ΔCBF in the SRMV group and ΔCBV in the MCA group had relatively high diagnostic accuracy and could be an optional predictor for people at high risk of CHS. CHS is more likely to occur when the ΔCBF of SRMV is greater than 95.04% or the ΔCBV of MCA is greater than 49.24%, although more large, prospective studies are needed to confirm and correct this result.

When the local hemodynamic changes generated by bypass surgery are excessive, impaired brain perfusion has been described, and can lead to neurological impairments and negative clinical consequences after surgery. SPECT, or PET, is indeed a commonly used method to examine cerebral perfusion. However, with the rapid development of imaging technology, magnetic resonance perfusion imaging has gradually become an important tool for hemodynamic monitoring of MMD. Arterial spin labeling (ASL) can be used to evaluate the collateral circulation perfusion in MMD. The measurements have been reported to be compatible with the gold standard approach^27^. Another study comparing SPECT and ASL imaging to measure cerebral blood flow in patients with cerebrovascular disease showed that ASL perfusion imaging can be used to determine cerebral perfusion status^28^. Due to its noninvasive technique, lack of need for the injection of exogenous contrast agents, non-radioactivity, and generally faster and less expensive advantages, MRI-ASL has gradually replaced SPECT, PET, and other invasive tests for the assessment of cerebral hemodynamics in patients with MMD^29,30^. Therefore, we used ASL to assess the cerebral perfusion status of patients before and after surgery. One of the severe side effects of MMD following revascularization is CHS, especially in adults, where the prevalence might reach 50%^31^. Age, hemorrhagic onset, low preoperative CBF values, a sharp increase in postoperative CBF, dominant hemisphere surgery, longer surgical time, and ineffective postoperative blood pressure management are specific factors that impact CHS^32^. The correlation between anastomotic hemodynamic changes around the anastomotic site and postoperative CHS has been reported^19,33^. Several papers have reported that CHS can result from excessive changes in hemodynamic changes caused by bypass surgery due to chronic ischemia, where the compensated vessels are initially in a state of vascular paralysis^20,34,35^. The main clinical manifestations of CHS are headache, transient aphasia, numbness and weakness in the contralateral limb, numbness in the contralateral face, and dysarthria, which often occur within a few days after the operation and can return to basic normal within 2 weeks^36^. The detection of CHS by imaging during the perioperative period is important in preventing serious postoperative complications. Current conventional cranial MR, CT, and other imaging examinations target postoperative brain tissue morphology, mainly to exclude postoperative cerebral hemorrhage or cerebral infarction, and have an indirect role in the diagnosis of CHS, which occurs mainly as a result of hemodynamic changes; therefore, the cerebral hemodynamic aspects of imaging are particularly important in the diagnosis of CHS^37^. In our study, changes in peri-anastomotic hemodynamics were compared before and after the procedure by applying ICG-FLOW800 video angiography, which can effectively respond to changes in the amount of local brain tissue perfusion.

ICG is a near-infrared fluorescent tri-carbon cyanide stain with a maximum absorption peak of 805 nm and the longest fluorescence peak of 835 nm. One of the energies after absorption is fluorescence. ICG cerebral angiography can be used during intracranial and extracranial vascular bypass surgery to observe the course of the graft, the presence of vessel collapse, distortion, and compression of the vessel by surrounding tissue, and to observe the patency of the penetrating branch vessels, supplying arteries, and distal vessels that have been revealed in the operative field^22,38^. The FLOW800 (Zeiss Meditec, Oberkochen, Germany) color fluorescence angiography is a technical upgrade on the ICG fluorescence angiography, which captures information on the parameters of ICG fluorescence angiography such as “transit time” and “fluorescence intensity” for post-processing analysis using the FLOW800 software integrated into the operating microscope. The FLOW800 software is post-processed and analyzed to provide a color map of the visual delay in hemodynamic and perfusion changes, allowing comparison of perfusion areas in the anastomosed region before and after bypass. The color codes for the color maps are determined by the sequence of the ICG contrast flow sites, which are color-coded in stages with the length of time passage of the ICG contrast. This color image is more visual, vivid, and provides more detail of the blood flow^24,25,39^.

ICG-FLOW800 video angiography clearly and visually characterizes the distribution of alternative recipient vessel populations in the surgical area and aids surgical decision-making and recipient vessel selection based on the evaluation of hemodynamic characteristics. Combined with the pre- and post-reconstruction hemodynamic characteristics, it provides guidance for the prediction of short-term postoperative neurological deficits and perioperative management^25,26^.

The majority of recent international studies on hemodynamic analysis before and after ICG-FLOW800 video angiography revascularization of MMD patients have concentrated on the thicker vessels surrounding the anastomosis, such as the STA and MCA^20,26^. In fact, our hemodynamic analysis of the peri-anastomotic area in MMD patients after bypass surgery is immediate with ICG-FLOW800 video angiography. However, we have found in our clinical work that the blood flow around the anastomosis is often in a state of hemodynamic disturbance such as turbulent or even reverse blood flow at this time^11,40^. We suspect that the hemodynamic parameters derived in this disturbed state are inaccurate. Similarly, the results of the analysis of the resulting parameters may not accurately represent the actual perfusion of brain tissue. As a result, we have shifted our ROI selection to the microvasculature. We believe that microvasculature, the “terminal vessels” that directly perfuse brain tissue, would appear to significantly reduce the influence of hemodynamics on brain perfusion.

Most international studies on cortical microvasculature in patients with MMD have focused on anatomical analysis. Czabanka et al. discovered a significant increase in cortical microvascular density (MD) and microvascular diameter in MMD patients, resulting in an increase in microvascular surface area (MVSA). In MMD patients, an increase in MVSA was positively connected with an increase in arterial microvascular transit time and negatively correlated with cerebrovascular reserve capacity^17,41^. Patients with greater MD exhibited more adequate collateral development and enhanced therapeutic outcomes after encephaloduroarteriosynangiosis, according to Wang et al.^42^. Relatively few studies have been reported on their hemodynamic relevance. CBF, TTP, and mean transit time (MTT) of microvascular were investigated by Kenji Uda et al. using FlowInsight software, but this method has not been widely used due to accessibility^19^. Haruto Uchino et al. applied ICG video angiography to analyze cortical vessels and predict CHS after direct bypass surgery in patients with MMD but did not perform a comparative analysis with other vessels^33^.

Therefore, we proposed the concept of SRMV and used the ICG-FLOW800 hemodynamic analysis software before and after bypass to select “rectangular” as the type of ROI pattern and to select the size of the ROI as “small”. The terminal branches of the vessels surrounding the recipient artery were selected as ROIs for hemodynamic analysis and analyzed in comparison with the thick recipient artery, thus adequately responding to the hemodynamic influence on the experimental results. Not only are there microvascular in SRMV as opposed to thick receptor arteries, which can greatly reduce the impact of hemodynamic disturbances on the analysis results, but there is also brain tissue that is directly perfused by the majority of microvascular. The actual perfusion of brain tissue can be more intuitively and realistically reflected by analyzing changes in fluorescence intensity of brain tissue, providing a more accurate analysis of the data obtained and yielding more accurate results for predicting the occurrence of postoperative CHS. In this study, we refined the study of intraoperative ROI selection in patients with MMD using ICG-FLOW800 video angiography by analyzing hemodynamic changes in SRMV, making changes in hemodynamic parameters in SRMV one of the effective predictors of postoperative CHS in MMD patients. In addition, a combination of fluid intake and output control, as well as strict monitoring of central venous pressure and blood pressure, is used to prevent CHS in advance, thus improving the clinical prognosis.

This research has certain limitations. Firstly, ICG-FLOW800 video angiography only measures hemodynamic factors in cortical areas of the brain and does not adequately reflect global blood flow. Secondly, differences in the rate and location of ICG injection may affect the values of each hemodynamic parameter, and fluctuations in blood pressure cannot be fully controlled. Finally, being a retrospective study is another limitation of this study. Factors such as small sample sizes and single-center studies may also affect the generalizability of the results. We believe that in the future, more advanced tools and multi-center tests with large samples will be available to further confirm or correct the findings of this research.

## Methods

### Patient date

A retrospective survey of the 36 cerebral hemispheres of 31 patients who underwent STA-MCA bypass at our institution from July 2021 to August 2022 was conducted. According to the presence or absence of CHS after surgery, CHS and non-CHS groups of patients were separated. One hemisphere (1 patient) had a new cerebral infarction after surgery, and on postoperative CT or MRI scans, none of the remaining patients had cerebral hemorrhage or infarction. Intraoperative hemodynamic characterization was performed using ICG-FLOW800 video angiography in all cases. The Chinese guidelines for the diagnosis and treatment of MMD and moyamoya syndrome developed by the Stroke Prevention Project Committee, National Health and Family Planning Commission, China, were followed by all patients. Prior to surgery, each patient had a magnetic resonance perfusion imaging to confirm areas of hypoperfusion. The study was approved by the Institutional Review Board of Qingdao University Hospital. Informed consent was obtained from the study participants, including legal guardians of minors.

### ICG-FLOW800 analysis and surgical procedures

A highly experienced chief neurosurgeon (Wei Liu) performed all procedures. The frontotemporal craniotomy was followed by an ICG-FLOW800 analysis. Select a recipient artery that is compatible with the diameter, direction of travel, and perfusion of the STA. ROI's maximum fluorescence intensity is used as the peak blood flow, which can be approximated as cerebral blood volume (CBV); the time to peak_1/2_ (TTP_1/2_) is used as the delay time; and the slope of the curve is used as the cerebral blood flow (CBF)^26^. The ROI is first set on the recipient artery and other surrounding thicker arterial branches, and hemodynamic parameters such as CBF, CBV, and TTP_1/2_ can be calculated. The ROI is then set on the SRMV, and the above hemodynamic parameters are again calculated. To check for revascularization and recalculate the hemodynamic parameters of the two ROIs mentioned above, the ICG-FLOW800 analysis was performed again following end-lateral anastomosis of the STA to the recipient artery.

### Definition of CHS and postoperative management

We defined CHS as intraoperative ICG angiography demonstrating a patent STA-MCA bypass conduit and an increase in CBF at the anastomotic location as seen on MRI-ASL on the first postoperative day. Within a few days, the patient had neurologic deficits like headache, transitory aphasia, numbness and weakness on the opposite side of the body, contralateral facial paresthesia, and dysarthria. After 2 weeks, the patient was back to almost normal. Other abnormalities, like a fresh postoperative cerebral infarction and surface-level temporalis muscle compression, were ruled out^15,36^. All postoperative patients have strict fluid intake and output controls. Central venous pressure and blood pressure are also strictly monitored. Cranial CT and MRI-ASL were performed on the first postoperative day to clarify the intracranial condition and cerebral blood perfusion. Within 2 weeks after surgery, if the patient develops new symptoms or the original symptoms worsen, a cranial CT or MRI is performed to exclude cerebral hemorrhage or cerebral infarction.

### Statistical analysis

According to the presence or absence of CHS after surgery, CHS and non-CHS groups of patients were separated. Depending on the type of ROI chosen, patients were separated into SRMV and MCA groups, and hemodynamic data from the SRMV and MCA groups were subjected to a paired samples t-test. The CHS group and the non-CHS group were compared using a t-test with independent samples. Mean ± standard deviation (SD) was used to express descriptive variables. The receiver operating curve (ROC) analysis was used to evaluate the predicted sensitivity and specificity of hemodynamic parameters. The area under the curve (AUC) with the greatest size that could be calculated using ROC analysis was used to measure diagnostic precision. Youden’s index analysis was applied to determine the ideal threshold for hemodynamic variables for predicting CHS. Database management and statistical analysis were performed by GraphPad Prism 9 and IBM SPSS Statistics 27 software. *p* < 0.05 was chosen as the statistical significance threshold. Specific data are detailed in the Supplementary material.

### Supplementary Information

Supplementary Information.

## Data Availability

The datasets for this study are protected patient information. Some data may be available for research purposes from the corresponding author upon reasonable request.
